# Implementation of Foundation Program under “Graduate Medical Regulations 2019” for first professional MBBS students at a Medical College located in western India - A transformative learning experience

**DOI:** 10.15694/mep.2020.000064.1

**Published:** 2020-04-03

**Authors:** Shobha Misra, Nilesh Fichadiya, Viren Kariya

**Affiliations:** 1 P D U Government Medical College

**Keywords:** Curriculum Reforms, Graduate Medical Regulations, Foundation Program, Curriculum Implementation Support Program

## Abstract

This article was migrated. The article was marked as recommended.

**Introduction**: Students enter new environment in medical colleges at around 17 years of age directly from school which can be challenging. Therefore, in Graduate Medical Regulations 2019 of India, attempt has been made to orient medical learners to MBBS program and provide them with requisite knowledge, communication, technical and language skills through a month-long foundation program. The purpose of this study is to share learning and to document feedback and best practices that would enhance the value and structure of the program in coming years.

**Methodology**: Descriptive evaluation of the foundation program implemented at a medical college located in western India as per the guidelines of the Curriculum Implementation Support Program (CISP). This program was implemented by all medical colleges under the ambit of Medical Council of India from August 2019.

**Teaching-Learning Methods**:Interactive sessions and assessment mainly based on reflective writing or by verbal/written feedback. To help in program evaluation and refinementa pretested semi-structured questionnaire administered to the students and faculty to gather their perceptions about various aspects of the course on a Likert scale of 1-5; 5-Strongly agree, 4-Agree, 3-Uncertain, 2-Disagree, 1-Strongly Disagree and three open-ended questions at the end of the course **.** Data was entered into Microsoft Excel 2007 and descriptive statistics utilized for interpretation of perceptions, themes and direct quotation used in the analysis.

**Take home Message**: The enthusiasm, hard work and integrated effort by the faculty members who participated in the program were extremely important for the success of this course. It is learnt that the Foundation program highlights benefits, is a valuable vehicle for increasing students’ overall confidence. There are challenges involved in operationalization viz; it requires more time and effort from faculty, at least in the initial phases of program development.

## Introduction

A curriculum defines the learning that is expected to take place during a course or programme of study in terms of knowledge, skills and attitudes. The written and published curriculum is the official or formal curriculum. Curriculum or course needs to be monitored and evaluated to ensure that it is working as planned and to identify areas or improvement.

The undergraduate medical curriculum of the Medical Council of India (MCI) has undergone revisions from time to time but the “Graduate Medical Regulations, 2019” represents the first major revision to the medical curriculum since 1997 and hence incorporates changes in science and thought over two decades ( MCI Document GMR, 2019). The curriculum is created to ensure that the medical doctor who emerges from the MBBS training program is capable of assisting the nation to achieve its goal of “Health for All” now modified as “Universal Health Coverage”. As part of the roadmap to the curricular roll out - a nationwide Curriculum Implementation Support Program ( MCI Document CISP, 2019) is being cascaded in a ‘train the trainer’ format hence, capacity building in the form of basic and advanced support for faculty is an ongoing activity of the MCI. The structural framework of support includes the Medical Education Unit (MEU) of the institutions and the Nodal and Regional Centers of the MCI. To these, the MCI has added the governance oversight of the curriculum in the form of the Curriculum Committee (CC) at the institutional level. The members of CC have the responsibility of training faculty of their respective colleges. The educators are required to impart transformative learning to create leaders in healthcare delivery system for the community. The thrust in the new Regulations is continuation and evolution of thought in medical education making it more learner-centric, patient-centric, gender-sensitive, outcome oriented and environment appropriate. The training is intense and demands greater commitment, resilience and lifelong learning. The revised curriculum was implemented by all medical colleges under the ambit of MCI in August 2019. The roll out of the overall curricular reforms will be progressive over the duration of the MBBS course.

Students enter new environment in medical colleges at around 17 years of age directly from school which can be challenging. Therefore, in the new curriculum attempt has been made to allow students from diverse educational streams and backgrounds in terms of geography, culture, language, economy, social construct, medium of instruction and education boards to transition appropriately from school to professional course through a foundation program ( MCI Document Foundation Course, 2019). This foundation program is of one-month duration, offered at admission to orient medical learners to MBBS program and provide them with requisite knowledge, communication (including electronic), technical and language skills. The purpose of this study is to share learning and to document feedback and best practices from the implementation of foundation program during August 2019. It is believed that this learning experience would enhance the value and structure of the program in the coming years.

## Methods

**Methodology**: As per the new curriculum guidelines (MCI Document: GMR, 2019) every year in the month of August a one-month foundation course needs to be implemented at the inception of first MBBS.

### Purpose of the Foundation Program/course are;

• To orient the undergraduate medical students to all aspects of the medical college environment.
• To equip them with certain basic, but important skills required for patient care and enhancing their communication, language, computer and learning skills.
• To provide them opportunity for faculty and peer interactions and an overall sensitization to the various learning methodologies.

It contains six modules like Orientation, Skills, Community orientation, Professionalism & ethics and Enhancement of language, computer skills and Sports and Extracurricular activities ( MCI Document Foundation Course, 2019). Each module has specific topic wise hour distribution with special hours allotted to sports and extracurricular activities in-between as shown in Table 1.

**Table 1.** Structure of the Foundation Program for the students

S. No	Themes/ subjects/contents	Total teaching hours
**1**	Orientation to medical profession, curriculum, institute, alternate health care system, society etc.	30
**2**	Skills Module-first aid, basic life support, bio safety etc.	35
**3**	Field visit to community health center.	8
**4**	Professional development including ethics.	40
**5**	Language/ computer skills	22
**6**	Sports and Extracurricular activities	40
	**Total teaching hours**	**175**

**Study setting & participants:** A month-long foundation program commenced on 1st of August 2019, at a Medical College located in western India. A batch of 200 students; 56 (28%) females and 144 (72%) males, took admission in the first professional course of MBBS, through an all India entrance test in the form of National Eligibility cum Entrance Test (NEET).

**Curricular Governance:** The governance oversight of the curriculum took place through the Curriculum Committee (CC) and the program was owned and conducted by the pre-clinical departments with appropriate input and faculty support by other departments. A foundation Program/Course Committee with a designated foundation program co-ordinator was formed under the principal of the Medical College in collaboration with the Curriculum Committee and MEU for smooth conduction of the foundation course. MEU undertook faculty development program to train and orient the resource persons. The curriculum committee co-ordinator in close collaboration with the foundation program committee members prepared a detailed schedule of the sessions for one month and allocated the sessions to the resource persons (including external resource persons from outside the college, if necessary). They closely monitored the program on a daily basis and coordinated with the administration, students and clinical faculty.

**Ethics:** Verbal consent was obtained from students and faculty before administration of questionnaire and use of photographs for research purpose. The protocol of the program was submitted to the Institutional Ethical Committee. The same was approved with no: PDUMCR/IEC/2376/2020, dated: 15/02/2020.

**Objectives of the implementation of foundation program were:** To gather perceptions of the students and faculty about various aspects of the course; to document feedback and best practices.

**Design:** The current study is a descriptive evaluation of the foundation program implemented at a government medical college located in western India. A detailed schedule was prepared well in advance showing all the aforementioned modules with their respective topics and as per the guidelines of the Curriculum Implementation Support Program ( MCI Document CISP, 2019 and MCI Document Foundation Course, 2019). On very first day a parent-student-faculty meet was organized and the session was inaugurated by the Principal of the college in presence of a spiritual leader to congratulate, motivate and bless the newly admitted students regarding the role of a doctor in the society and they were appraised of institute and university rules & regulations and anti-ragging environment of the institute. On subsequent days, internal & external guest faculty interacted with the students regarding doctor patient relationship, role of a doctor in the society and the role of an Indian Medical Graduate (IMG).

The students were divided into small batches and were then taken for hospital visit and community health center visit accompanied by faculty from community medicine department. Before visiting these centers, the students were appraised on various National Health Policies and Healthcare System of India. Simultaneously, in rotation these batches undertook stress management & computer skills training. There were detailed sessions on professionalism and ethics module; viz interpersonal relationships, professional and altruistic behavior, time management, learning strategies, disability competences, organ and dead body donation awareness, ragging etc. Dedicated time slots were allocated for local (Gujarati) and English language skills for the students joining from outside Gujarat and from Gujarati medium respectively. They were separate sessions on communication skills and basic computer skills as per the schedule. There was protected time slot given to the students for reflective writing/journal to reflect on their learning of various sessions.

To conduct and to complete the entire foundation program effectively and as per schedule a total of 60 faculty contributed their time and efforts. Out of which, 8 were invited as guest faculty from various specialties, who were awarded certificate of appreciation. Others were the internal faculty from various departments of the institute.

**T-L Methods:** The shift in focus from knowledge acquisition to application necessitates important changes in the learning process: Therefore, GMR, 2019 lays great emphasis on (a) shared responsibility in the learning process (b) self-directed and collaborative learning. Dedicated time for self-directed learning is provided in each subject in every phase (c) use of learner centric approaches (d) skill acquisition and certification e) formative assessment as integral to the learning process (f) progressive increase in the complexity of learning (the so-called ascendancy in competencies). Didactic lectures should not exceed one third of the schedule; two third of the schedule should include interactive sessions, practical, clinical or/and group discussions. The learning process should include living experiences, problem-oriented approach, case studies and community health care activities.

**Figure 1.**
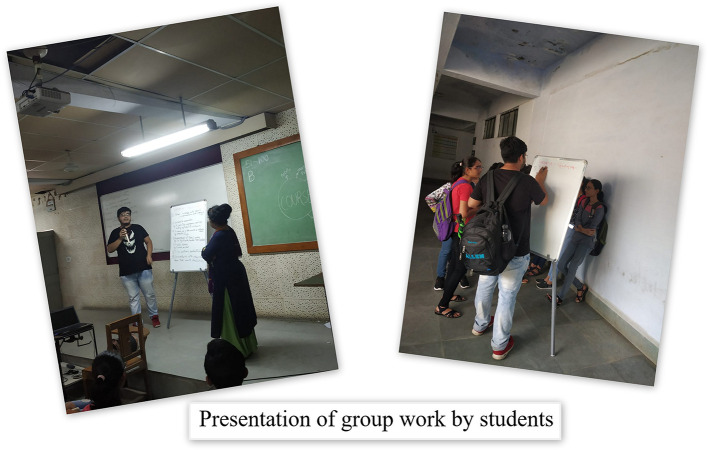
Presentation of group work by students

**Figure 2.**
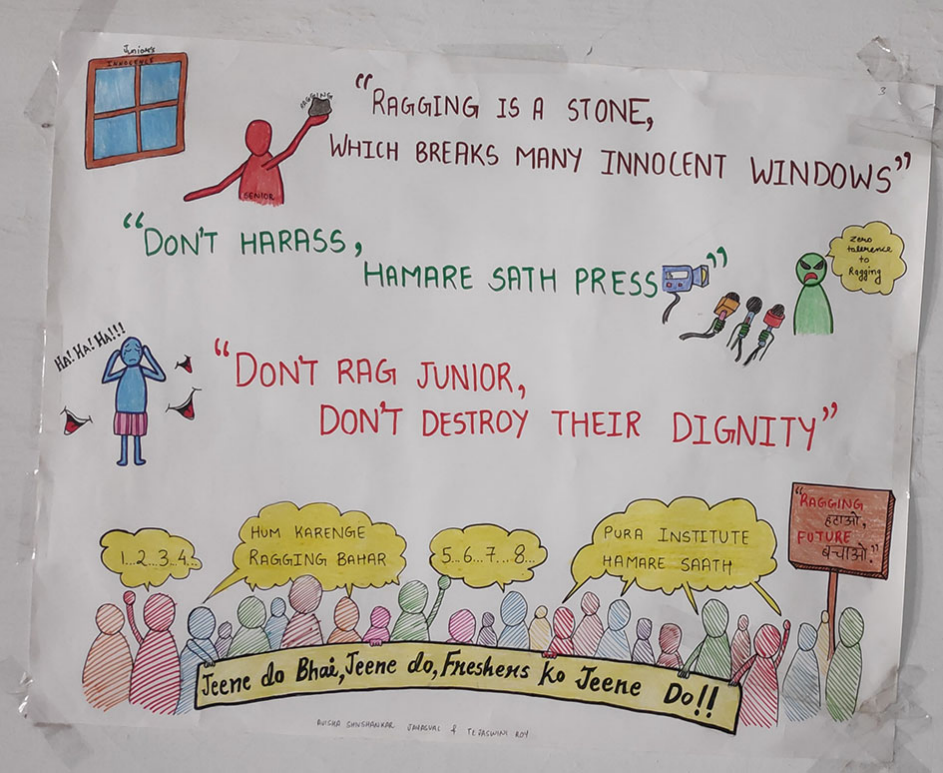
Poster on “anti-ragging” by student

**Figure 3.**
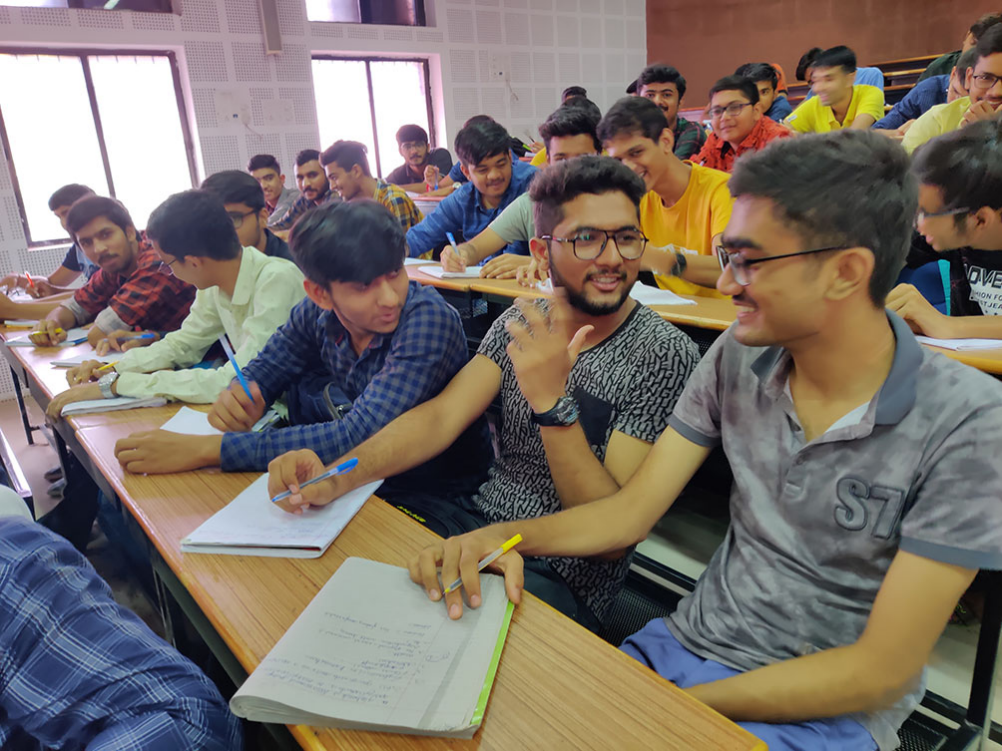
Think-pair-share - A teaching-learning method

**Figure 4.**
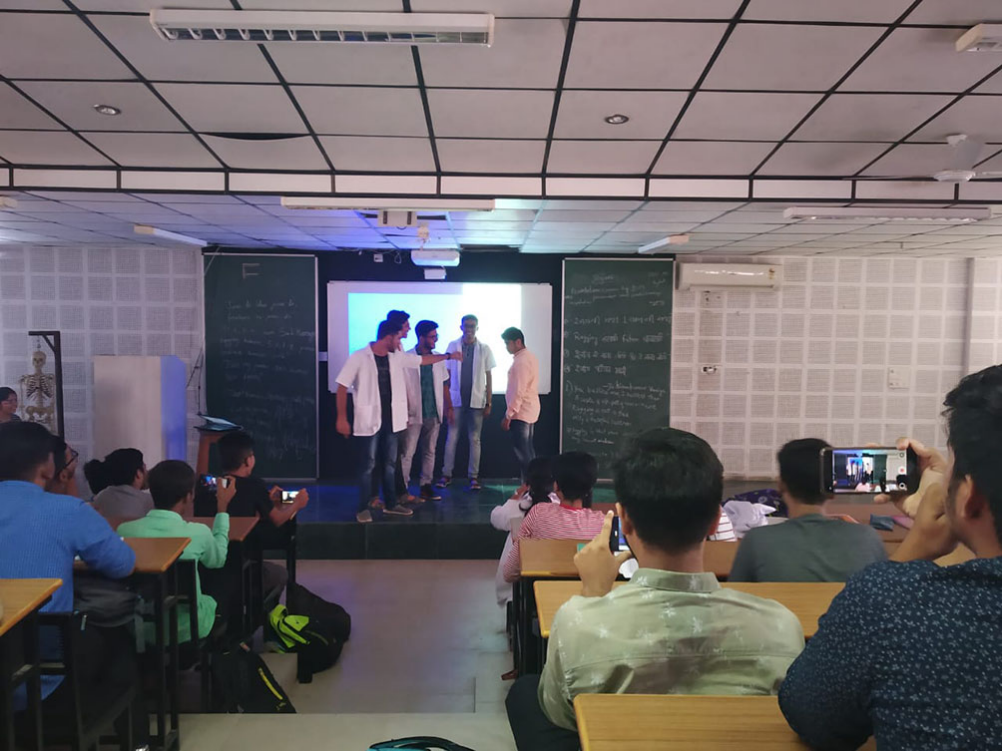
Role play on “anti-ragging” by students

Therefore to motivate students to develop the habits of self-directed learning, greater emphasis in the foundation program was laid on student engagement techniques viz; symposia, seminars, panel discussion, small group discussions with gallery walk, problem-oriented and problem-based discussions assisted by videos, poster and slogan preparation, drama, role play, sports and games, think pair share, visit to hospital, visit to community health centres and collaborative and team based learning as shown in Figures 1- 4. A poster and slogan competition carried out on a theme of “anti-ragging” turned out to be very innovative and informative as shown in Figure 2.

**Formative and Internal assessment**: Foundation program is compulsory under the new GMR and an attendance of 75% is essential, hence this criterion was conveyed to the students on the very first day of their joining the program. Throughout the program a complete record of attendance was kept to maintain punctuality and regularity. 96 % of the students met the criteria of 75% attendance. As per the GMR the performance of the students in the foundation program shall not contribute towards internal assessment marks. However, the students completed reflective writing journals to reflect on their learning as part of formative feedback assessment to the students and the faculty. Voluntary verbal feedback was obtained on the last day of the course by four students of both genders. On the last day of the program, a pretested, semi-structured questionnaire was administered to the students and faculty to gather their perceptions about various aspects of the course on a Likert scale of 1-5; 5-Strongly agree, 4-Agree, 3-Uncertain, 2-Disagree, 1-Strongly Disagree and three open-ended questions to help in program evaluation and refinement. The data was entered into Microsoft Excel 2007 and descriptive statistics utilized viz: Mean, SD and Confidence Interval for quantitative data and % for qualitative data. For interpretation of perceptions themes were analyzed and direct quotation are used in the analysis.

## Results/Analysis

### Program Evaluation

The revised curriculum “Graduate Medical regulations 2019” was introduced for the first time and implemented by all medical colleges under the ambit of MCI in August 2019. A batch of 200 students; 56 (28%) females and 144 (72%) males, took admission in the first professional course of MBBS at Medical College in western India in August 2019. We describe the design, implementation and evaluation of one-month long foundation program, the students undertook after admission. Out of the 200 students in the class, 189 were present on the last day and filled the evaluation questionnaire. And out of the 52 internal faculty involved from different department, 18 of them completed the same.

### (A) Evaluation by the Students

**Table 2.** Score given by students for various aspects of the program (Max.: 5, Min.:1)

Sr No	Statement	Mean (SD)	95% CI
**1**	Foundation program was an enjoyable learning experience	4.06 (0.80)	2.49 - 5.63
**2**	An effective program to make one realize “What the profession of doctor is”?	4.60 (0.56)	3.48 - 5.67
**3**	Various activities and discussions had a stimulating effect on one’s feelings / emotions	4.00 (0.76)	2.46 - 5.37
**4**	It helped know peers, faculty and new environment.	4.30 (0.75)	2.83 - 5.78
**5**	Shall enhance the ability to communicate with peers and teacher due to teaching of communication skills	4.00 (0.81)	2.34 - 5.51
**6**	Shall enhance the ability to manage time better	4.02 (0.78)	2.48 - 5.55
**7**	Shall enhance ability to deal with stress effectively	4.00 (0.93)	1.97 - 5.61
**8**	Most of the students were given opportunity to get actively involved in the program	4.23 (0.87)	2.51 - 5.93
**9**	The learning environment was comfortable and relaxing in most of the sessions	4.00 (0.89)	2.08 - 5.58
**10**	The teachers involved in conduct of sessions were knowledgeable and conducted the sessions well	4.38 (0.76)	2.89 - 5.88
**11**	The teachers were very approachable and gave freedom to ask questions	4.60 (0.67)	3.27 - 5.91
**12**	We had freedom to express fully during various activities of the program	3.70 (0.87)	1.98 - 5.39
**13**	The speakers spoke the language that was easily understood	4.00 (1.09)	1.75 - 6.05
**14**	The time managed by the speakers was appropriate	3.73 (0.95)	1.86 - 5.59
**15**	Course is necessary to begin with, as it provided knowledge and a strong foundation for my medical studies and career as doctor	4.52 (0.76)	3.03 - 5.998
**16**	Decreased fear/tension and increased overall confidence as a medical student	4.07 (0.81)	2.488 - 5.65
**17**	Need of assessment / exam at the end of the course to check what has been learnt.	2.00 (1.15)	-0.34 - 4.16

SD: Standard Deviation, CI: Confidence Interval

The mean score by students to various aspects of the program on a scale of one to five as shown in Table 2 is above average (4 for majority of the aspects) except for the “Need of Assessment” which reinforces that there is no need for formal assessment in this program.

**Table 3.** Summary of findings about perceptions of students for various aspects of the course

Areas with good feedback (strengths)	Areas that need improvement (weaknesses)
Increased Students’ confidence	Technical problems causing inconvenience viz, audio visual aids
Enhanced knowledge on topics: viz Basic Life Support (BLS), Biomedical Waste Management (BMW), Immunization, Documentation, etc.	Inadequate infrastructure; lecture hall being small to accommodate 200 students, no availability of water at the time of session
Role clarity on 5 roles of IMG, viz: professionalism, lifelong learner, leader, communicator, clinician	Non availability of outdoor games facilities viz, playground, cricket kit, sports equipment, sports teacher
Oriented about Professionalism & Ethics	Some lectures were found to be boring
Improved skills for stress management and time management	Language barrier to AIQ (All India Quota) students to communicate in vernacular (Gujarati) language
Appraised about the importance of Yoga and Alternative Medicine	
Good exposure to hospital setting through visits and observation and exposed to community level health centers viz; Urban Health Center where opportunity was given to communicate with the patients and their relatives	
Sports and extra-curricular activities were enjoyable and destressing	
Group activities were enjoyable and interesting	
Knowing each other increased the comfort level to express self in front of the class	

Table 3 summarizes the perceived strengths and weaknesses of the program by the students. Students’ reflections in their own words are listed in Table 4 as Quotes.

**Table 4.** Students’ Quotes

“I am very fortunate to attend this program of foundation course. I would like to thank every faculty who took session. It was really helpful to increase my confidence, knowledge, belief to my profession, mental abilities. It was really helpful to visit community health center which increased our communication ability. However, some of the technical problem caused inconvenience during sessions and sometimes the required things were not available.”
“I suggest the future students to attend all the sessions of foundation program as it helps a lot.”
“We learnt to communicate with people in society because of foundation program.”
“Field visits made our perspective clearer about practicing as doctor, different type of patients and how to deal with them, perception of people about a doctor and we also realized our responsibility.”
“It was a great opportunity to express ourselves in front of all during foundation program.”
“We learnt very essential & basic skills from this foundation course. It was well organized. I learnt to develop a good personality, leadership and professionalism which is very important in the career of a doctor. It will be very much useful for us in our future. I learnt how to interact with my peers, teachers, patients and with other people. It will improve my communication skill also.
“I was able to gain confidence on myself. I began to love myself. I face my fears, one of which was speaking on stage. I participated in activities in which I thought I was never capable of. I made very good friends through the way of one month. It was amazing.”
“Hospital visits, urban healthcare center visits were undoubtedly the best part of the program. They allowed us to engage freely and obtain hands on experience about out health care system.”
“The most beautiful thing was that I overcome my own fear not fully but I did. I faced 200 students watching at me and I said what I learnt and what I felt in sessions. So, it was very good experience.”
“Foundation course was excellent as per my opinion. Our faculty did all the things very smoothly and nicely. I am very luck and thankful.”

**(B) Evaluation by the Faculty;** Almost all of the 18 faculty who completed the feedback questionnaire rated the various aspects of the program as good to very good (between 3 to 5 of 5). Major strength of the program was that; the students were thoroughly interested, the program provided them a common platform and enhanced coordination between different faculty. Some of the suggestions for improving the program were;need toinvolve a greater number of external faculty from specific subjects like language (English, vernacular- Gujarati), sports, computer, advance planning, greater co-ordination among faculty, optimum and adequate resources, duration of didactic lecture be made shorter.

One of the faculty put it like this, “the strength of this program is that students are aware about the whole MBBS curriculum but the challenge is that resource persons from outside needs to be invited”.

One another said “what I observed was, some students were thoroughly interested while some were not. So, extra efforts should be made to make every student interested in the program. Also, that the sessions should be made short and sweet”.

## Discussion

***Lessons learnt:*** It is learnt that the Foundation Program highlights benefits, is a valuable vehicle for increasing students’ overall confidence. The program benefits as perceived by the students included; helping them to gain clarity on their future roles as medical professional/doctor as envisaged by MCI, orienting them to medical ethics and professionalism, enhancing their time and stress management skills, enhancing their communication, language, computer and learning skills, oriented them towards alternative systems of medicine, exposure to hospital and community health centers provided opportunity to communicate with the patients and their relatives leading to realization of the authentic setting of their future role, orienting them towards importance of sports and extracurricular activities to mention a few. Based on faculty feedback, this provided a common platform and enhanced coordination between different faculties. We also learned that there are challenges involved in operationalization viz; it requires more time and effort from faculty, at least in the initial phases of course development. Other challenges include; finding ways to include a greater number of external faculty from specific subjects like language, sports and computer skills, sound planning and co-ordination among faculty along with strong will of those involved, optimum resources in terms of infrastructure for sports, dedicated vehicle for visits to community health centers and audiovisual aids. Inclusion of in-depth qualitative feedback from faculty and students would yield better insight into scaling of the program.

## Conclusion

To summarize, we learned that this type of foundation program is feasible within a conventional medical curriculum. The benefits of such learning include clarity on future roles as medical professional/doctor as envisaged by MCI right from intake into the program, thus improving knowledge and skills of learners, and hence likely to improve quality of health care provided to the patients.

**Limitations:** One of the limitations of this study is that it evaluated single medical school, which may not represent the “Indian Medical Graduate” as a whole. Therefore, caution should of course be exercised in extrapolating results to all students in all medical schools. Further studies should aim to determine attitudes of medical students in all medical schools of India to get higher generalizability.

## Take Home Messages

• The enthusiasm, hard work and integrated effort by the faculty members who participated in the program were extremely important for the success of this program.

• It is learnt that the Foundation Program highlights benefits, is a valuable vehicle for increasing students’ overall confidence. The benefits of such learning include clarity on future roles as medical professional/doctor right from intake into the program, thus improving knowledge and skills of learners, and hence likely to improve quality of health care provided to the patients.
• There are challenges involved in operationalization viz; it requires more time and effort from faculty, at least in the initial phases of course development. Other challenges include; finding ways to include a greater number of external faculty from specific subjects like language, sports and computer skills, sound planning and co-ordination among faculty along with strong will of those involved, optimum resources in terms of infrastructure for sports, dedicated vehicle for visits to community health centres and audio-visual aids.
• We learned that this type of program is feasible within a conventional medical curriculum.

## Notes On Contributors

**Dr. Shobha Misra** is working as Professor & Head at department of community medicine at P D U Medical College, Rajkot. She holds degrees in community medicine, naturopathy & yoga, hospital administration, mother & child health, human resource management. She is FAIMER fellow from Philadelphia & has been honoured with F-IAPSM. She has a teaching experience of 28 years. She is actively involved in teaching & training of health professionals. She has 40 publications in various national & international journals to her credit.

**Dr. Nilesh Fichadiya** is working as lecturer in department of community medicine at P D U Medical College, Rajkot. He is involved in teaching undergraduate professional MBBS students for last 10 years. He is MD in community medicine and diploma in public health. He is also working as program incharge for certificate course in community health being conducted at community medicine department.

**Dr. Viren Kariya** is working as Associate Professor in department of Anatomy at P D U Medical College, Rajkot for last 15 years. He was the program coordinator for the program.
