# Supplementary material for: Implementation of Foundation Program under “Graduate Medical Regulations 2019” for first professional MBBS students at a Medical College located in western India - A transformative learning experience
Source: MedEdPublish (2016). 2021 Sep 27;9:64. Originally published 2020 Apr 3. [Version 2] doi: 10.15694/mep.2020.000064.2 (PMC10697521; doi:10.15694/mep.2020.000064.2)
Supplement: Peer review history

# Reviewer report: Reviewer response for version 2

**DOI:** 10.21956/mep.20265.r31451

**Published:** 2021-10-11

**Authors:** Richard Hays

**Affiliations:** 1 James Cook University

This review has been migrated. The reviewer awarded 3 stars out of 5 Student contributions are always welcome and this is no exception. The concept of a nation-wide, 4 week-long foundation program for entering medical students is interesting. I believe that most medical schools have programs that ease new students into what is often very intense study, but in most cases I have seen this is usually within the first 1-2 weeks. For secondary school leavers this can be a steep learning curve to different modes of learning with greater autonomy and responsibility. Hence this description is useful, showing how a slower, planned process engages new students. What leapt out at me in the data were a suggestion that the program may have been a little too teacher-centric, with a lot of faculty driven content in lectures. Perhaps more student-run activities with more senior students would add values? The take home messages show that the faculty are thinking about making improvements. Overall this is well worth reading by those engaged in Year 1 course management.

Reviewer Expertise:

NA

No decision status is available

# Reviewer report: Reviewer response for version 2

**DOI:** 10.21956/mep.20265.r31452

**Published:** 2021-10-05

**Authors:** Ken Masters

**Affiliations:** 1 Sultan Qaboos University

This review has been migrated. The reviewer awarded 4 stars out of 5 I am pleased to see that the authors have addressed the major concerns I raised with Version 1 of their paper. The Discussion, though, is still very weak, and the authors have missed the opportunity for this study to be more solidly placed within the literature on the topic. (That said, some of the material they placed into the Results does serve this purpose, but it would have been more appropriate to place it in the Discussion).Nevertheless, the paper is a useful read and contribution to the field. Thank you.

Reviewer Expertise:

NA

No decision status is available

# Reviewer report: Reviewer response for version 1

**DOI:** 10.21956/mep.19133.r27633

**Published:** 2020-06-11

**Authors:** Ken Masters

**Affiliations:** 1 Sultan Qaboos University

This review has been migrated. The reviewer awarded 3 stars out of 5 The paper deals with student and staff perceptions towards the introduction of a Foundation program at a medical school in order to meet changes in guidelines. Overall, the study has been well-conducted. There are, however, areas that need to be addressed.• Introduction: Apart from the local administrative documentation, there is no literature referenced. The problem with doing that is that it confines the importance of the research to a very localised context only. If the authors wish the paper to have interest to a wider audience, then they need to broaden their literature – at the very least, they need to refer to academic literature dealing with these issues in India, by going beyond the three pieces that they have cited.• Results: I would like to see the qualitative data laid out more formally according to themes (Identification of theme, followed by 2-3 supporting quotations). At the moment, the qualitative data are simply deposited on the page, with no real context.• The Discussion is weak with, again, no reflection on the results in light of current literature. If the authors leave it as it is, then it remains a paper with very localised interest only. The authors need to be able to relate their results to current literature on the topic.Minor• There are many small language errors, especially in dropping articles, and I would encourage the authors to carefully proof-read their manuscript before re-submission.• The term “ragging” as it appears to be used in this paper, is not universally known, and should be explained.So, while the study is worthwhile, the authors have done themselves a disservice by not viewing their study within the broader context. I look forward to a Version 2 in which this is corrected.

Reviewer Expertise:

NA

No decision status is available

# Reviewer report: Reviewer response for version 1

**DOI:** 10.21956/mep.19133.r27632

**Published:** 2020-04-13

**Authors:** Puja Dulloo

**Affiliations:** 1 Pramukhswami Medical College

This review has been migrated. The reviewer awarded 3 stars out of 5 Interesting article on the foundation program as per the revised competency based undergraduate medical curriculum drafted by Medical Council of India (MCI).Introduction, methodology, and analysis of data have been well explained. My suggestions for intensification of the manuscript are follows:It would have been best to obtain written consent at the beginning of the study once ethical approval was acquired by the institutional ethical authority, since you have presented students pictures, their poster presentation and role play.Validation method of questionnaire has been described. Why this questionnaire was not filled by all the students if everyone had undergone the program (being mandate as per council guidelines). Out of 60 faculty members only 18 filled the feedback form, no specific reason has been highlighted by the author.The authors can compare this program with similar programs globally or it would have been good to compare it with the orientation program being conducted by various Indian medical institutes for previous batches. How this program is better than the earlier orientation program? Quotes from the students could generate a theme thus present as a qualitative analysis of the presented data.The authors need to have continuous focus on this batch so as to identify attitudinal modification of the students as they move ahead.All the best for further analysis of the study.

Reviewer Expertise:

NA

No decision status is available

# Reviewer report: Reviewer response for version 1

**DOI:** 10.21956/mep.19133.r27634

**Published:** 2020-04-04

**Authors:** P Ravi Shankar

**Affiliations:** 1 American International Medical University

This review has been migrated. The reviewer awarded 4 stars out of 5 This is an interesting article on the foundation program at a medical school in western India. The authors have put in a lot of effort into designing and conducting the program and in carrying out the study. The study is well described and the methodology is clear. My suggestions for further strengthening the manuscript follow:The authors can mention the process followed for selecting medical students in India and mention that most students join the program after twelve years of schooling. The authors have obtained ethical approval for the study but the fact that they obtained only verbal consent may raise some ethical issues. This is especially true as they have also used identifiable student photographs in the article. The same is also true with regard to the participation of faculty members. Where, how and when were the questionnaires administered? The participation of the faculty was low while I believe all students who were present on the final day of the session participated in the study. Where were the sessions held? One challenge would be accommodating the large number of students (200). From the photographs it seems like most sessions were held in lecture halls. The proportion of female students in the college is low. What is the relationship between the MEU and the curriculum committee? The authors in the Discussion can try to compare the foundation program with other similar programs globally though the duration and the methods employed may be different. All educators, especially those from India, will find this article to be of interest.

Reviewer Expertise:

NA

No decision status is available
